# 18 GHz electromagnetic field induces permeability of Gram-positive cocci

**DOI:** 10.1038/srep10980

**Published:** 2015-06-16

**Authors:** The Hong Phong Nguyen, Yury Shamis, Rodney J. Croft, Andrew Wood, Robert L. McIntosh, Russell J. Crawford, Elena P. Ivanova

**Affiliations:** 1 School of Science, Swinburne University of Technology, Melbourne, Australia; 2 Illawarra Health and Medical Research Institute, Wollongong, Australia; 3 Australian Centre for Electromagnetic Bioeffects Research, Australia; 4 School of Health Sciences

## Abstract

The effect of electromagnetic field (EMF) exposures at the microwave (MW) frequency of 18 GHz, on four cocci, *Planococcus maritimus* KMM 3738, *Staphylococcus aureus* CIP 65.8^T^, *S. aureus* ATCC 25923 and *S. epidermidis* ATCC 14990^T^, was investigated. We demonstrate that exposing the bacteria to an EMF induced permeability in the bacterial membranes of all strains studied, as confirmed directly by transmission electron microscopy (TEM), and indirectly via the propidium iodide assay and the uptake of silica nanospheres. The cells remained permeable for at least nine minutes after EMF exposure. It was shown that all strains internalized 23.5 nm nanospheres, whereas the internalization of the 46.3 nm nanospheres differed amongst the bacterial strains (*S. epidermidis* ATCC 14990^T^~ 0%; *Staphylococcus aureus* CIP 65.8^T^ *S. aureus* ATCC 25923, ~40%; *Planococcus maritimus* KMM 3738, ~80%). Cell viability experiments indicated that up to 84% of the cells exposed to the EMF remained viable. The morphology of the bacterial cells was not altered, as inferred from the scanning electron micrographs, however traces of leaked cytosolic fluids from the EMF exposed cells could be detected. EMF-induced permeabilization may represent an innovative, alternative cell permeability technique for applications in biomedical engineering, cell drug delivery and gene therapy.

Cell permeability is the route by which the passage of different types of exogenous ions, molecules/macromolecules can pass through the cell membrane^1^,^2^,^3^,^4^,^5^. This process is of substantial significance in biomedical engineering, drug delivery and gene therapy applications^1^,^5^,^6^,^7^,^8^,^9^. One of the proposed causes of cell permeability is the formation of pores on the bacterial cell membrane, which is often called ‘membrane poration’^1^,^2^,^3^,^4^,^5^. Cell membrane poration is known to be caused by the rearrangement of the molecular structure of the membrane, together with interactions taking place at the aqueous–lipid interface, which can be physically induced through the application of external shocks such as mechanical stress^10^,^11^, ultrasound (sonoporation)^2^,^9^,^12^, electric fields (electroporation)^1^,^3^,^5^,^6^,^13^,^14^,^15^, and laser (photoporation or optoporation)^4^,^16^,^17^,^18^,^19^. Inducing pores in the membrane is believed to lead to the relaxation of the surface tension of the membrane^20^, together with a consequent change in the osmotic pressure that is due to the passage of internal and external components through the pores that are formed^1^,^13^,^20^. The initial rupture of the membrane leads to the formation of cylindrical pores that become lined with phospholipid head groups; these continue to increase in size until a state of zero surface tension is reached^11^,^20^. Such membrane pores can either be temporary, resealing after a given elapsed period, or continue to expand and eventually rupture the membrane. The effect that results is dependent on the degree of external shock, duration of exposure, and the characteristics of the cells under consideration^6^,^15^,^21^.

It was recently reported that exposing *Escherichia coli* cells to EMFs at 18 GHz (with a resultant temperature of 40 °C) caused the internalization of large macromolecules such as dextran (150 kDa)^22^,^23^,^24^. It was suggested that in contrast to low-frequency and traditional EMF exposures, high-frequency EMFs amplify and enhance the electro-kinetic processes, and do so without damaging the cells^22^,^25^,^26^. Since it remains unclear as to whether other bacterial taxa with different cell wall structures and compositions to that of Gram-negative *E. coli*^27^,^28^,^29^,^30^,^31^,^32^ (*e*.*g*., Gram-positive bacteria) would be affected in a similar way, the aim of this study was to investigate whether the application of EMF exposures at the microwave (MW) frequency of 18 GHz would induce permeability in the membranes of Gram-positive cocci; *Planococcus maritimus* KMM 3738, *Staphylococcus aureus* ATCC 25923, *S. aureus* CIP 65.8^T^ and *S. epidermidis* ATCC 14990^T^. In this work, propidium iodide^13^,^33^, large (23.5 nm and 46.3 nm) silica nanosphere uptake assays, Confocal Laser Scanning Microscopy (CLSM), TEM and SEM were employed to assess whether the cells could be made permeable under certain carefully defined experimental conditions. We also aimed to determine the size of the polymer nanocarriers that can be delivered into the cytosol via this method.

## Results

### EMF induced permeability in Gram-positive coccoid bacterial cells

**Figure 1:**
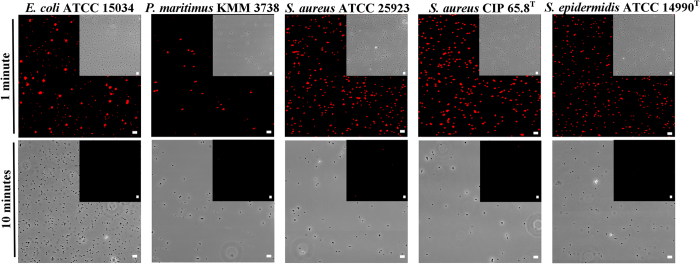
Propidium iodide internalization by the bacterial cells after EMF exposure. CLSM images showing propidium iodide internalization after 1 min (first row) following EMF exposure. Phase contrast micrographs showing bacterial cells in the same field of view. No propidium iodide was observed in any tested cell types after 10 min (same field of view insets in the second row). Scale bars in all fluorescence images are 5 μm.

**Figure 2:**
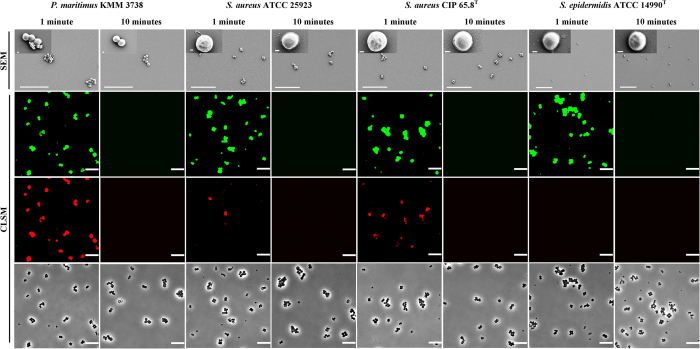
Bacterial cell response to EMF exposure. Typical scanning electron micrographs of *P. maritimus* KMM 3738, *S. aureus* ATCC 25923, *S. aureus* CIP 65.8^T^ and *S. epidermidis* ATCC 14990^T^ cells at 1 min and 10 min after EMF exposure. No significant change of cell morphology was observed (insets). Scale bars are 10 μm, inset scale bars are 200 nm. CLSM images showing intake of 23.5 nm nanospheres (second row) and 46.3 nm nanospheres (third row), 1 min after EMF exposure. After 10 min both types of nanospheres were not able to be internalized by the cells. The phase contrast images in the bottom row show the bacterial cells in the same field of view. Scale bars are 5 μm.

**Figure 3:**
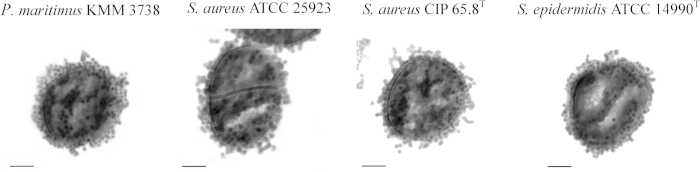
Nanosphere (23.5 nm) internalization by the bacterial cells following EMF exposure. Typical TEM images of thin-sectioned (80 nm) cells of *P. maritimus* KMM 3738, *S. aureus* ATCC 25923, *S. aureus* CIP 65.8^T^ and *S. epidermidis* ATCC 14990^T^ showing the uptake of 23.5 nm nanospheres. Scale bars are 200 nm.

The CLSM analysis of EMF exposed bacterial cells showed that the EMF induced membrane permeability of the cells of the four Gram-positive strains tested (*E. coli* was used as a reference strain, Supplementary Fig. S1), as confirmed by the uptake of propidium iodide (Fig. 1 and Supplementary Fig. S1). It was also found that approximately 97% ± 5% of *P. maritimus*, 99% ± 4% of *S. aureus* ATCC 25923, 99% ± 3% of *S. aureus* CIP 65.8^T^ and 99% ± 5% of *S. epidermidis* cells were able to internalize 23.5 nm silica nanospheres (Fig. 2). A similar effect was observed for the *E. coli* cells (98% ± 4%) (Supplementary Fig. S2). CLSM analysis indicated that the internalization of the nanospheres could continue for up to approximately 9 min after the initial EMF exposure (data not shown), whereas no uptake of the nanospheres was detected when the bacteria were exposed to the nanospheres 10 min after the EMF exposure (Fig. 2). The same bacterial cells in the respective control groups were subjected to conventional heating, replicating the temperature profiles of the cells being subjected to the EMF exposure (using a Peltier plate, Supplementary Fig. S1 and S3). These reference group cells were not able to internalize the propidium iodide, however, it should be noted that up to approximately 5% of these reference cells were observed to be capable of internalizing nanospheres, most likely due to the presence of damaged or dead cells, which are often present in cell populations^34^. TEM analysis confirmed the uptake of 23.5 nm-nanospheres by the EMF exposed cells (Fig. 3). Within the cross-sectioned cells, it can be seen that some of the nanospheres were located around the cell membrane and others within the cells themselves, whilst the majority were found in the cytosol. In contrast, non-EMF exposed cells remained intact (Supplementary Fig. S4), with the majority of the cells (95%) showing no internalisation of either form of nanosphere.

While all of the strains tested were found to internalize the 23.5 nm-nanospheres, the extent of uptake of the 46.3 nm-nanospheres was variable amongst the strains studied (Table 1). For example, a large proportion of the *P. maritimus* cells (80% ± 9%), and almost half of the *S. aureus* ATCC 25923 and *S. aureus* CIP 65.8^T^ cells (40% ± 7% and 44% ± 7%, respectively) were able to internalize these nanospheres, however none of the *S. epidermidis* cells were able to do so.

The nanosphere loading capacity of a single bacterium was evaluated for each of the bacterial strains studied (Table 1). It was found that up to approximately 261 of the 23.5 nm nanospheres and 114 of the 46.3 nm nanospheres could be internalized by a single coccoid cell after exposure to EMF.

### EMF effect on cell morphology

The SEM analysis of EMF exposed bacterial cells did not reveal any significant change in the morphology of the cocci; however, the some traces of leaked cytosolic fluid could be seen surrounding the cells of each strain studied (Fig. 2). The morphology of the non-treated and Peltier heat-treated reference cells were also unchanged (Supplementary Fig. S3).

### Effect of EMF on cell viability

**Figure 4:**
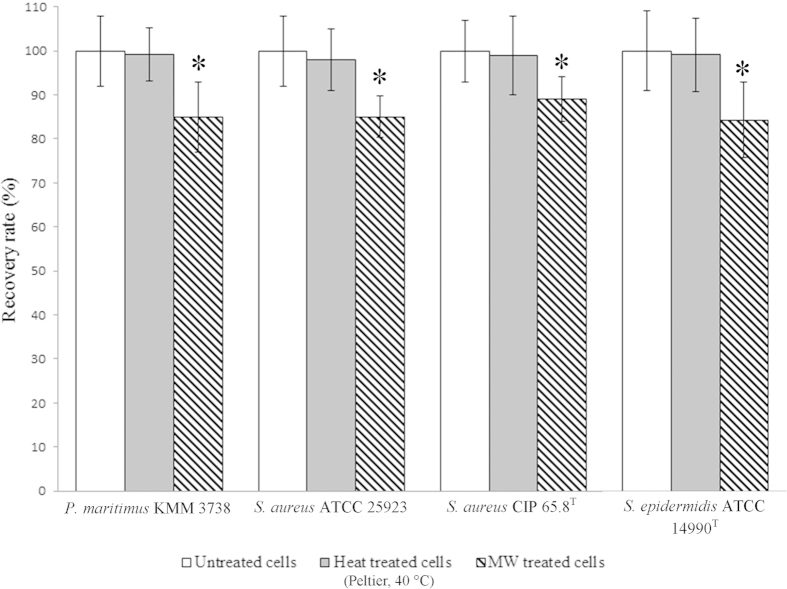
Effect of EMF exposure and bulk heat on the bacterial cell viability. The recovery rate (%) of EMF exposed cells (shaded white), Peltier heated 40 °C (grey) and untreated cells (white). Cells inactivated by boiling (100 °C) were non-viable. Data are means ± standard deviation (SD) and representative of 3 independent experiments with 10 replicates each. *p < 0.05 versus the corresponding controls.

Cell viability experiments were performed via the direct counting of colony forming units and were conducted for the EMF exposed, Peltier plate heated and non-treated cells. The results showed that above 84% of each of the strains studied (85% ± 8% *P. maritimus*, 85% ± 5% *S. aureus* ATCC 25923, 89% ± 5% *S. aureus* CIP 65.8^T^ and 84% ± 9% of the *S. epidermidis* cells) remained viable after the EMF exposures (Fig. 4). The Peltier plate heated cells maintained their viability (99% ± 6% *P. maritimus*, 98% ± 7% *S. aureus* ATCC 25923, 99% ± 9% *S. aureus* CIP 65.8^T^ and 99% ± 8% of the *S. epidermidis* cells). A statistical analysis of the data did not reveal a statistically significant difference between the viability of the Peltier heated and untreated cells (*P. maritimus* (*p* > 0.05), *S. aureus* ATCC 25923 (*p* > 0.05), *S. aureus* CIP 65.8^T^ (*p* > 0.05) and *S. epidermidis* (*p* > 0.05)) (Fig. 4). Although the cell viability of EMF exposed cells was only slightly affected, this difference was found to be statistically significant in comparison to the controls (p < 0.05).

## Discussion

The results reported here provide evidence that the exposure of cocci to EMF at 18 GHz, with a specific energy absorption rate (SAR) value approximately 5.0 kW kg^−1^, induced cell permeability in each strain being investigated. The SAR was determined experimentally in this work because it has been suggested that it is a more accurate estimation energy absorption for biological material^33^,^35^,^36^,^37^, because the variations in specific heat within biological matter are usually much smaller than corresponding variations in conductivity, resulting in a much more uniform temperature than electric field distribution^35^,^36^,^37^. It is of interest to note that certain biological effects of SAR values in the range of 4.0 kW kg^−1^ at 8.53 GHz and 4.85 kW kg^−1^ at 2.45 GHz, have been described, e.g., three-dimensional conformational changes in green fluorescent protein (GFP)^25^ and increased citrate synthase binding efficiency^26^, respectively; however, no comparable data are available regarding bacterial cells.

Here, the induced permeability of bacterial cells was confirmed by propidium iodide intake, as well as TEM and CLSM microscopy which allowed visualization of the internalized nanospheres. The propidium iodide assay has been applied as the standard technique used for confirming electroporation of bacterial membranes^13^,^33^. Propidium iodide does not normally pass through intact membranes^13^,^33^, however, when a cell membrane is disrupted, the propidium cation (Pr^2+^) can pass through the membrane and bind to the nucleic acids within the cell, which will eventually fluoresce^33^. Further examination of CLSM and TEM micrographs revealed that indeed after exposure to an 18 GHz EMF, the bacterial cells were capable of internalizing both propidium iodide and nanospheres as a direct consequence of membrane traffic modulation (Figs. 1, 2, 3). The consistent internalization of both propidium iodide and nanospheres was observed in different cocci species, despite the variations in cell wall structure between species^38^,^39^. Since no internalisation of either propidium iodide or nanospheres was observed for the bacterial cells subjected to conventional heat treatment (Peltier plate heating, Supplementary Fig. S1-S4), it can be assumed that the EMF-induced cell permeabilization could not be attributed simply to the bulk temperature rise of the cells, and therefore must have arisen as a direct result of either the interaction of EMF with the bacterial cell membrane and its components (*e.g*., phospholipids, membrane proteins, etc), or microthermal changes not detectable at the macro level.

The great efficiency (97%) with which the bacteria populations that were exposed to EMF were able to internalize 23.5 nm nanospheres is an important characteristic; for example, the efficiency of the photoporation-induced permeability has been reported to be 85–100% with up to 80% cell viability^4^, and the permeabilization efficiency of sonoporation reported to be 78% with 82% cell viability^2^. Furthermore, a pore lifetime of 9 min appears to be comparable with that obtained using electroporation or photoporation processes^40^. For example, Saulis *et al.* reported that under the influence of a single electric field pulse, human red blood cells were able to be permeabilized (allowing the internalizing of ascorbic acid and mannitol), but that the membrane barrier function partially recovered after 3 min and that complete resealing of the pores was attained after 10 min^40^. Similarly, Schneckenburger *et al.* employed the focused beam of an argon ion laser (488 nm) to photoporate Chinese hamster ovary (CHO-K1) cells^41^. These authors reported the formation of small circular black spots of approximately the same size as the focused beam, which disappeared within about 5 min^41^.

We believe, however, that the nature of the permeabilization that arises from the exposure of the bacterial cells to EMF at 18 GHz is very different to that in previously-reported pore formation phenomena^1^,^3^,^4^,^13^,^14^,^18^,^19^,^22^,^42^: Electroporation induces pore formation in the cells that are exposed to an electric field^1^,^6^,^14^,^15^,^43^, during which, cells have to be placed in close proximity between two electrodes. The presence of an electric field changes the electrochemical potential across the cell membrane, locally inducing instabilities through the formation of defects in one of the leaflets of the membrane^1^,^6^,^7^,^14^,^15^,^44^. Photoporation occurs when tightly focused laser light is used to induce the reversible poration of the cellular membrane, allowing exogenous materials to enter the cell^4^,^16^,^17^,^18^,^19^,^41^. Laser light with wavelengths in the ultraviolet (UV), visible (VIS), and infrared (IR) ranges, both as pulsed (ns or fs) and continuous waves (CW), have been used for photoporation^19^. For CW lasers, the poration mechanism would most likely occur as a result of localized heating of the cellular membrane by the laser irradiation^4^,^16^,^41^. It is thought that high frequency electromagnetic fields, through their resultant high frequency vibrations, generate an external mechanical stimulation to the cell membrane^45^. The modulation of the latter results in enhanced membrane trafficking via exocytosis/endocytosis, as was reported, for example, for several (eukaryotic) cell types^45^,^46^. Other studies, including Karshafian *et al.*, reported the presence of sonoporation-induced membrane-pore like defects of the murine fibrosarcoma cell line KHT-C^2^. These authors estimated the ultrasound-induced pore size (in the presence of micro-bubbles) to be in the range of 20 nm to 500 nm, based on their ability to internalize different molecular weight markers (10 kDa to 2 MDa FITC-dextran)^2^. Similarly, Zhou *et al.* reported that the ultrasound-induced pores of the *Xenopus laevis* oocyte cell membrane were of a diameter in the order of 220 nm (with a standard deviation of 80 nm, due the changes of the trans-membrane current (TMC) of a single cell under voltage clamp)^47^. The size of the exogenous materials internalized into eukaryotic cells by exposure to an electric field of a similar field strength or ultrasound was reported to be much smaller (*i*.*e*., less than 6 nm^1^,^2^,^3^,^4^,^13^,^14^,^41^,^42^,^48^), however, no direct confirmation of the actual pores formed in the plasma membrane were presented in these studies.

The present findings suggest that the mechanical stimulation of cellular membranes resulting from exposure to the high frequency vibrations resulting from exposure to 18 GHz EMF radiation changes the membrane tension, causing it to deform, inducing an endocytosis-like process in the bacterial cell walls. Endocytosis is an endomembrane dynamic feature of eukaryotes that has not been previously reported for bacteria, with the exception of recently discovered subcellular compartmentalization in two bacterial taxa, *Planctomycetes* and *Verrucomicrobia*^49^,^50^,^51^,^52^,^53^. For example, Lonhienne *et al.* have shown an endocytosis-like green fluorescence protein (GFP) being taken up by *Gemmata obscuriglobus* bacterial cells^53^. Our results thus raise the possibility that EMF might induce subcellular compartmentalization. The variation in the bacterial cells’ ability to internalize the 46.3 nm nanospheres might be due to the differences in cell wall structure and/or the phospholipid composition of the cell membranes of different bacteria. For example, it is well documented that *Staphylococcus* species vary in the type of teichoic acids present, one of the essential components of the peptidoglycan in the Gram-positive cell wall^54^,^55^,^56^. Endl *et al.* reported that poly(glycerolphosphate) teichoic acids are characteristic components of the *S. epidermidis* peptidoglican, whereas poly(ribitolphosphate) teichoic acids are characteristic of the *S. aureus* cell wall^55^. It may be speculated that due to the differences in the molecular weight and three-dimensional organization of glycerol and ribitol molecules, the ability to internalize nanospheres may have been affected to such an extent that the *S. epidermidis* cells were unable to internalize the 46.3 nm nanospheres. In contrast, the *P. maritimus* cells showed a greater propensity for the internalization of 46.3 nm nanospheres (80% susceptible cells). *P. maritimus* is a member of the genus *Planococcus,* which represents a bacterial lineage of peculiar irregular morphology^57^, adopting a coccoid-like shape due to being Gram-variable motile cocci^58^,^59^, and characteristic cell-wall structure whose chemical composition is yet to be determined.

To the best of our knowledge, this is the first time the physical internalization of large polymeric carriers and biomolecules by bacterial cells via an endocytosis-like process has been reported. We hypothesize that the modulation of the membrane permeability, induced by the high-frequency vibrations induced by 18 GHz EMF, is electro-kinetic in nature due to the resulting increased conductivity, diffusion and ion mobility induced in the cell membrane^24^,^40^,^41^. EMF-induced permeabilization may represent an innovative alternative cell permeability technique for applications in biomedical engineering, cell drug delivery and gene therapy^1^,^6^,^7^,^8^,^9^. Further studies are required to investigate the 18 GHz EMF-induced permeability in eukaryotic cells and to elucidate the mechanism/s by which EMF interacts with the microbial cell walls and/or cell membranes.

## Materials and methods

### Bacterial strains, cultivation procedures and sample preparation

Four strains of coccoid bacteria, *P. maritimus* KMM 3738, *S. aureus* ATCC 25923, *S. aureus* CIP 65.8^T^, and *S. epidermidis* ATCC 14990^T^, were studied. *E. coli* ATCC 15034 was used as a reference strain. Bacterial strains were obtained from the American Type Culture Collection (ATCC, Manassas, VA, USA), the Culture Collection of the Pasteur Institute (CIP, Paris, France) and the Collection of Marine Microorganisms (KMM, Vladivostok, Russian Federation). All strains were routinely grown on nutrient agar (NA, Oxoid, Basingstoke, England) or marine agar (MA, Becton Dickinson). Prior to each experiment, the *Staphylococcus* spp. strains were grown overnight at 37 °C and *P. maritimus* grown at 25 °C. All strains were collected at their stationary phase of growth (as confirmed by growth curves, data not shown) in order to utilize mature cells for the experiments^34^. Working bacterial suspensions were freshly prepared for each independent experiment. The cell density was adjusted to OD_600_ = 0.1 in phosphate buffered saline (PBS), 10 mM, pH 7.4, using a spectrophotometer (Dynamica Halo RB-10 UV-Vis, Precisa Gravimetrics, Dietikon, Switzerland).

### EMF exposure

**Figure 5:**
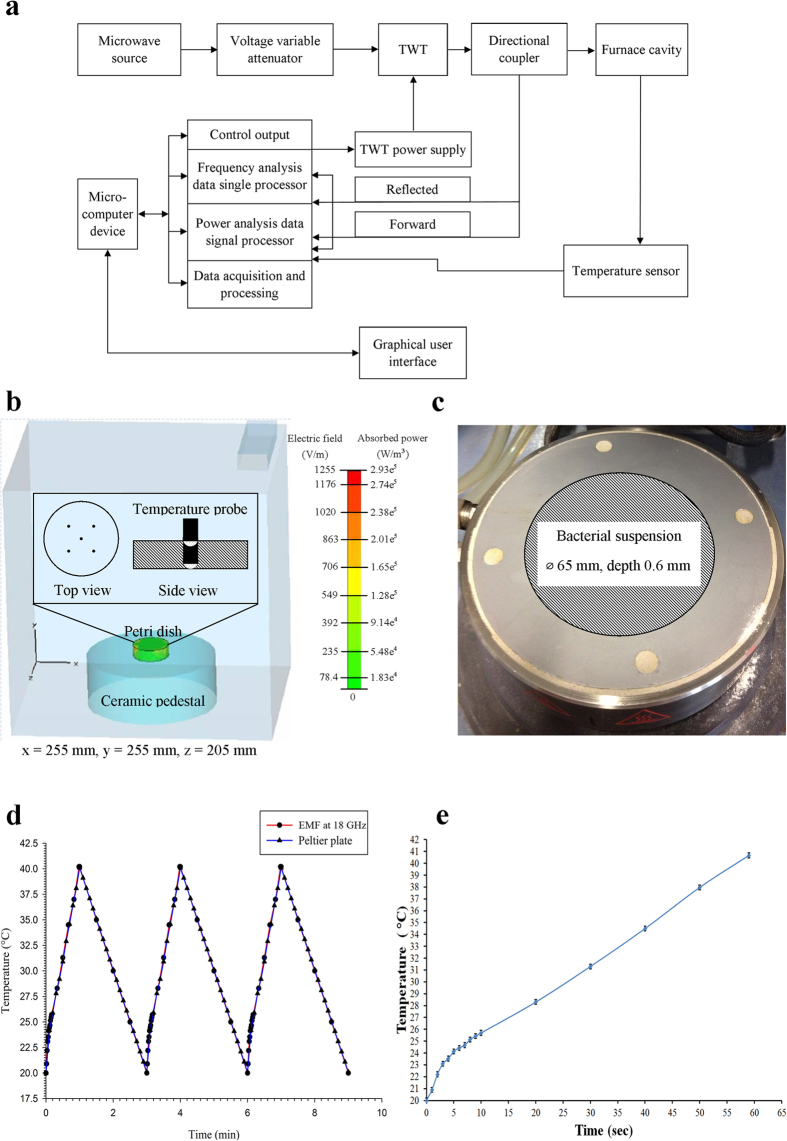
EMF configuration schematic and Peltier heating dynamics during treatments (MW and Peltier plate). (**a**) Schematic diagram of the EMF system; (**b**) Electric field and absorbed power modeling using CST Microwave Studio 3D Electromagnetic Simulation Software and inset images of the tip positions of the temperature probe in media (top and side view); **(c)** Peltier heating stage (diameter = 65 mm, suspension depth = 0.6 mm) with position used for applying the bacterial suspension; (**d**) The temperature profiles of bacterial suspensions during three consecutive EMF exposures and Peltier Plate heating/cooling control; (**e**) The heating rate of the bacterial suspension: increasing at a rate of approximately 1 °C per s during first 3 s and then reducing to a rate of approximately 0.3 °C per s for the remainder of the time.

The samples for EMF exposure comprised of 2 mL of bacterial cells suspension in a micro Petri dish (35 mm diameter, Griener Bio One, Frickenhausen, Germany). The EMF apparatus used for all experiments was a Vari-Wave Model LT 1500 (Lambda Technologies, Morrisville, USA) (the EMF configuration is shown in Fig. 5a) with a fixed frequency of 18 GHz using the settings detailed elsewhere^22^.

Each sample was subjected to three consecutive EMF exposures (resulting in a temperature increase in the samples ranging from 20 °C to 40 °C at a heating rate of 20 °C per min) for 1 minute, allowing the sample to cool to 20 °C on ice (at a rate of 10 °C per minute) between exposures. In order to obtain a uniform temperature gradient and avoid “hot spot” effects, the samples were placed onto a ceramic pedestal PD160 (Pacific Ceramics, Sunnyvale, CA, USA, ε’ = 160, loss tangent < 10^−3^) within the same position that had been identified, using electric field modelling using CST Microwave Studio 3D Electromagnetic Simulation Software (CST MWS) (CST of America, Framingham, MA, USA), as being the position that provided the most consistent heating environment (Fig. 5b). Since the average dielectric constant of the bacterial suspension was assumed to be that of water at EMF frequencies of 18 GHz, the dielectric loss tangent describing the energy dissipation was also assumed to be that of water at 25 °C and 18 GHz. The calculated wavelength of the EMF in water was determined to be 2.34 mm, which is greater than the dimensions of each bacterial cell. The depth of penetration was calculated to be 1.04 mm, which is also greater than the thickness of the bacterial suspension in the Petri dish. Hence, the possibility of subjecting the samples to non-even heating due to the presence of a non-uniform field distribution was considered negligible. The bulk temperature rise of the bacterial suspension was monitored via a built-in temperature probe, a Luxtron Fiber Optic Temperature Unit (LFOTU) (LumaSense Technologies, Santa Clara, CA, USA), of which the tip was placed at various positions (0 and 0.5 mm from the bottom of the plate) in the Petri dish, as shown in Fig. 5b. According to the manufacturer’s specifications, the tip of the probe is less than 1 mm thick and operates with an accuracy of ± 0.2 °C and 250 ms (in water) response time. The temperature was also carefully monitored after EMF exposure and during cooling using a portable Cyclopes 330S infrared/thermal monitoring camera (Minolta, Osaka, Japan). A total of 60 measurements were collected from 10 positions in the Petri dish, which included five different locations and two different depth levels within the bacterial suspension). It was found that the temperature variation was only in the range of ± 0.2 °C. The temperature profiles are reproduced in Fig. 5d and the heating rate arising from the EMF exposure is shown in Fig. 5e.

The SAR, given in kW kg^−1^, was calculated in the medium using Equation 1:


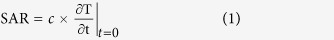


where *c* is the specific heat capacity of the medium (kJ kg^−1^ °C^−1^), and 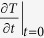 is the time derivative of the temperature determined at t = 0 s (°C s^−1^).

For the 10 measurement locations, the SAR was calculated using Equation 1 to be 5.0 ± 1.3 kW kg^−1^. It was assumed that the specific heat capacity of the bacterial suspension was the same as that of water at 25 °C, which is 4.18 kJ kg^−1^ °C^−1^.

The liquid evaporation that took place during the EMF exposures was found to be approximately 1.5% using an analytical balance (Cheetah Scientific, France) and therefore regarded as negligible.

### Bulk heat treatment

A Peltier plate heating/cooling system (TA Instruments, New Castle, DE, USA) was used to replicate the bulk temperature profiles during the EMF exposures according to the method previously described^22^. Briefly, a 2 mL volume of bacterial suspension was applied directly onto the Peltier plate sample platform (Fig. 5c), and subjected to heating from 20 °C to 40 °C for 1 min, followed by cooling to 20 °C for 2 min between heating treatments. The diameter of the Peltier plate sample platform was 65 mm and the bacterial suspension layer thickness was calculated to be 0.6 mm. All Peltier plate heated samples were performed in triplicate, and in parallel with EMF exposure experiments. The bulk temperatures during heating and cooling were monitored using the portable infrared/thermal monitoring camera Cyclopes 330 S (Fig. 5d). Working bacterial suspensions that were not exposed to either EMF exposures or heat treatment were used as the negative controls for all experiments.

### Scanning Electron Microscopy (SEM)

A field emission scanning electron microscope FeSEM – SUPRA 40VP (Carl Zeiss, Jena, Germany) with a primary beam energy of 3 kV was used. A 100 μL aliquot of bacterial suspension was placed on a glass cover slip (ProSciTech, Kirwan, Australia) in duplicate for each condition. After one min, the glass cover slips were washed with nanopure H_2_O (with resistivity of 18.2 MW cm^−1^), dried with 99.99% purity nitrogen gas, then exposed to gold sputtering (6 nm thick gold film) using a NeoCoater MP-19020NCTR (JEOL, Tokyo, Japan). The remainder of the bacterial suspensions, which were left to stand for 9 and 10 min, were subjected to the same treatment. Approximately ten SEM images were taken at 5,000 × and 70,000 × magnifications for each sample and analyzed.

### Confocal Laser Scanning Microscopy (CLSM)

Propidium iodide (1.0 mg mL^−1^ solution in water; Life Technologies Australia, Mulgrave, Australia) was used at a concentration of 500 nM to determine the viability of the bacterial cells^33^. Non-treated cells, cells inactivated by boiling (100 °C) and heat-treated cells using the Peltier plate (40 °C) were used as three different types of control. Heat inactivated cells were prepared by boiling the bacterial suspension for 15 min, followed cooling in a 25 °C water bath for 30 min. PI was added to all the bacterial suspensions at 1 min and 10 min after EMF exposures. The PI remained in contact with the bacteria throughout the experiment.

Two types of fluorescent silica nanospheres 23.5 ± 0.2 nm (FITC) and 46.3 ± 0.2 nm (Rhodamine B) (Corpuscular, Cold Spring, NY, USA) were added to the bacterial suspensions at a concentration of 15 μg mL^−1^ after periods of 1, 9 and 10 min following EMF exposures and heat treatment. The controls for these experiments comprised an untreated bacterial suspension that was mixed with the fluorescent silica nanospheres and Peltier heated bacterial suspensions mixed with the fluorescent silica nanospheres using the Peltier plate up to 40 °C. These were processed in parallel with the EMF exposed bacterial suspensions. Each suspension was then washed twice, followed by centrifugation at 4500 rpm for 5 min. The supernatant was then removed and re-suspended in PBS. A 100 μL aliquot of each sample was then analyzed using a Fluoview FV10i-W inverted microscope (Olympus, Tokyo, Japan). Approximately 5 CLSM images per treatment group were obtained, with each containing at least 50 bacterial cells per image.

### Transmission Electron Microscopy (TEM)

Bacterial suspensions with the 23.5 nm nanospheres were pelleted by centrifugation at 4800 rpm for 5 min at 25 °C. The cells were washed twice with PBS to remove all the unbound nanospheres. The pellets were suspended in 2 mL of 4% glutaraldehyde in PBS (10 mM, pH 7.4) for 30 min. The cells were then washed twice in PBS for 5 min. After the final washing step, the cells were mixed thoroughly in 0.5 mL of 5% agarose gel by stirring. The agar was immediately cooled to 4 °C by refrigerating for 30 min, then cut into 1 mm^3^ cubes and fixed with 1 mL of 1% osmium tetroxide (OsO_4_) for 1 h. The cubes were washed twice in nanopure H_2_O (with resistivity of 18.2 MW cm^−1^) for 15 min. The cells were dehydrated by passing the cubes through a graded ethanol series (20%, 40%, and 60%) (2 mL) for 15 min and stained for 8 h with 2% uranyl acetate in 70% ethanol (2 mL). After staining, the bacterium was further dehydrated by passing the cubes through another graded ethanol series (80%, 90% and 100%) for 15 min (2 mL)^60^,^61^.

The embedding medium was prepared from araldite, dodecenyl succinic anhydride (DDSA) and benzyldimethylamine (BDMA) (ProSciTech) and stir thoroughly^62^. In order to embed, each cube was washed twice with 100% acetone (2 mL) for 20 min, then incubated in 2 mL of acetone and embedding medium (1:1 ratio) for 8 h, followed by transfer to acetone and embedding medium (1:3 ratio) for 8 h and finally transfer into pure embedding medium for 8 h. The cube was transferred to embedding mould containing fresh pure embedding medium, which was then polymerized for 24 h at 60 °C^63^. The final block was trimmed and cut into ultrathin sections (80 nm thickness) with Leica EM UC7 ultramicrotome (Leica Microsystems, Wetzlar, Germany) and glass knife. Sections were put on 200 mesh copper grids and examined in JEM 1010 (JEOL).

### Quantification of internalized nanospheres

The nanosphere loading capacity of the four EMF exposed bacterial strains was quantified according to the fluorescence intensity of internalized silica nanospheres. A POLARstar Omega microplate reader (BMG Labtech, Ortenberg, Germany) was used to measure the fluorescence intensity of nanospheres in each bacterial suspension (Supplementary method). Each sample was prepared according to the method used for CLSM analysis.

### Cell Viability

A 100 μL volume of *Staphylococcus* spp. suspension was spread onto NA and a 100 μL volume of *P. maritimus* was spread onto MA; the *Staphylococcus* spp. were incubated for 24 h at 37 °C and the *P. maritimus* was incubated for 48 h at 25 °C. Cells inactivated by boiling (100 °C) were also examined. All the cell viability tests were conducted right after treatments and in parallel. Ten plates were used for each type of treatments in three independent experiments for each sample, to allow a statistical analysis to be performed. Three independent Student t-tests were performed to compare the consistency of decontamination rates across conditions, experiments and bacterial test strains.

All statistical data processing was performed using SPSS 21.0 software (SPSS, Chicago, IL, USA).

## Additional Information

**How to cite this article**: Nguyen, T. H. P. *et al.* 18 GHz electromagnetic field induces permeability of Gram-positive cocci. *Sci. Rep.* **5**, 10980; doi: 10.1038/srep10980 (2015).

## Supplementary Material

Supplementary Information

## Figures and Tables

**Table 1:** Internalization of silica nanospheres by bacterial cells following EMF exposures.

	**Silica nanospheres 23.5** **nm**		**46.5** **nm**	
**Bacterial strains**	Loading capacity*	**Bacterial cells that internalised nanospheres(%)**	Loading capacity*	**Bacterial cells that internalised nanospheres (%)**
*P. maritimus* KMM 3738	172 ± 8	97 ± 5	75 ± 8	80 ± 9
*S. aureus* ATCC 25923	161 ± 8	99 ± 4	81 ± 8	40 ± 7
*S. aureus* CIP 65.8^T^	261 ± 8	99 ± 3	114 ± 8	44 ± 7
*S. epidermidis* ATCC 14990^T^	211 ± 8	99 ± 5	Not detected	Not applicable

^*^per single bacterium

Nanosphere loading capacity was calculated using the fluorescence intensity of nanospheres. The number of bacterial cells that were able to internalize the nanospheres, expressed as a percentage, was calculated by counting fluorescent cells in the CLSM images. Data are means ± standard deviation (SD) and are representative of 3 independent experiments.
