# Erratum: 18 GHz electromagnetic field induces permeability of Gram-positive cocci

**DOI:** 10.1038/srep13507

**Published:** 2015-09-02

**Authors:** The Hong Phong Nguyen, Yury Shamis, Rodney J. Croft, Andrew Wood, Robert L. McIntosh, Russell J. Crawford, Elena P. Ivanova

*Scientific Reports* **5**: Article number: 1098010.1038/srep10980; published online 06 16 2015; updated: 09 02 2015.

This Article contains an error in Affiliation 4. The correct affiliation is listed below:

School of Health Sciences, Swinburne University, Melbourne, Australia

