# Supplementary material for: 18 GHz electromagnetic field induces permeability of Gram-positive cocci
Source: Sci Rep. 2015 Jun 16;5:10980. doi: 10.1038/srep10980 (PMC4468521; doi:10.1038/srep10980)

**18 GHz electromagnetic field induces permeability of Gram-positive cocci**

The Hong Phong Nguyen^1^, Yury Shamis^1^, Rodney J. Croft^2,3^, Andrew Wood^1,3^,

Robert L. McIntosh^1,3^, Russell J. Crawford^1^ and Elena P. Ivanova^1,3*^

^1^ School of Science, Swinburne University of Technology, Melbourne, Australia

^2^Illawarra Health and Medical Research Institute, Wollongong, Australia

^3^Australian Centre for Electromagnetic Bioeffects Research, Australia

*Corresponding author mailing address: School of Science, Swinburne University of Technology, PO Box 218, Hawthorn, Vic 3122, Australia; Telephone: +61392145137; Facsimile: +61398190834; e-mail: eivanova@swin.edu.au

**SUPPLEMENTARY DATA**

A calibration curve was constructed to determine the correlation of fluorescent intensity and nanosphere concentration. A total of seven nanosphere concentrations were prepared (0.005, 0.05, 0.5, 1, 5, 19 and 15 µg mL^-1^).

The mass of a silica nanosphere was determined using Equation 2.

$m=\rho\times V$ (2)

where *m* is the mass of a silica nanosphere (g), *ρ* is the mass density of silica (g cm^-3^) and *V* is the volume of a silica nanosphere (cm^-3^).

The volume of a silica nanosphere can be calculated using Equation 3.

$V=\frac{4}{3}\pi r^{3}$ (3)

where V is the volume of a silica nanosphere (cm^3^) and r is the radius of a silica nanosphere (cm).

The radii of the 23.5 nm and 46.3 nm nanospheres were 11.75 × 10^−7^ cm and 23.15 × 10^−7^ cm, (Corpuscular), volume 6.8 × 10^−18^ and 5.2 × 10^−17^ cm^3^, and mass 1.8 × 10^−17^ and 1.38 × 10^−16^ g, respectively. The mass of a single nanosphere was used to calculate the number of internalized nanospheres.

The concentration of internalized 23.5 nm nanospheres was found to be approximately 0.31 µg mL^-1^ for *P. maritimus*, 0.29 µg mL^-1^ for *S. aureus* ATCC 25923, 0.47 µg mL^-1^ for *S. aureus* CIP 65.8^T^ and 0.38 µg mL^-1^ for *S. epidermidis* cells. By dividing the concentration of the nanospheres by the total cell concentration in the bacterial suspension (10^8^ cells mL^-1^), the mass of the internalized nanospheres was found to be approximately 3.1 fg cell^-1^ (*P. maritimus*), 2.9 fg cell^-1^ (*S. aureus* ATCC 25923), 4.7 fg cell^-1^ (*S. aureus* CIP 65.8^T^) and 3.8 fg cell^-1^ (*S. epidermidis*) and the number internalized nanospheres by a single bacterium was 172 spheres cell^-1^ (*P. maritimus*), 161 spheres cell^-1^ (*S. aureus* ATCC 25923), 261 spheres cell^-1^ (*S. aureus* CIP 65.8^T^) and 211 spheres cell^-1^ (*S. epidermidis*). The number of 46.3 nm internalized nanospheres was similarly quantified.

**SUPPLEMENTARY FIG. S1| Propidium iodide internalization by the control groups**. No propidium iodide uptake was observed after heat treatment using Peltier plate (first row) and untreated cells (inset images of bottom row). Cells inactivated by boiling (100 °C) allowed the propidium iodide to be taken into the cells (second row). Scale bars in all fluorescence images are 5 μm.


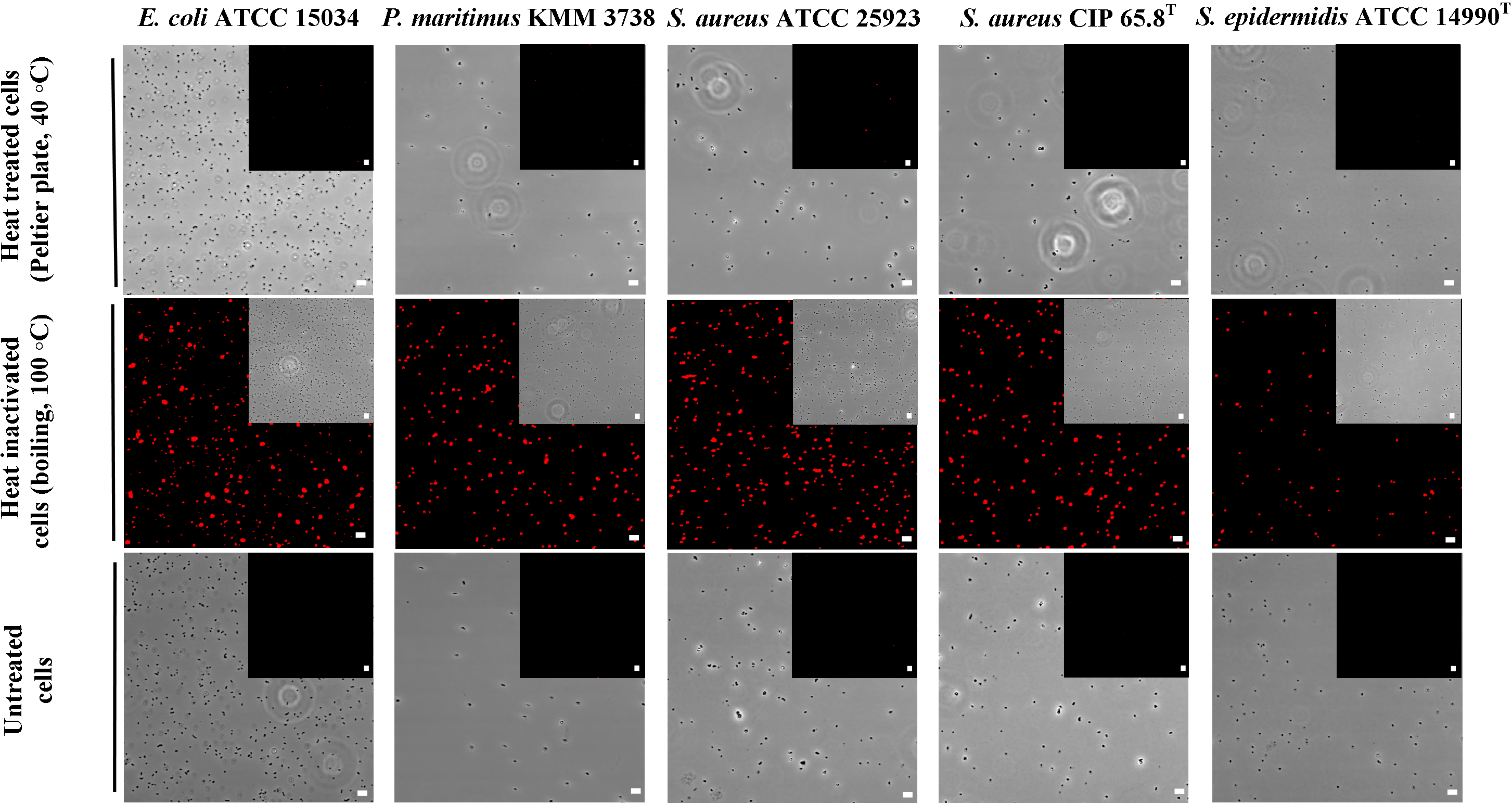


**SUPPLEMENTARY FIG. S2| EMF effect on *E. coli***. CLSM images confirming the internalization of 23 nm nanospheres (top row, green) and 46 nm nanospheres (bottom, red), 1 min after EMF exposures. Approximately 10 min after EMF exposures, no uptake of the nanospheres could be observed. Scale bars in all fluorescence images are 5 μm.


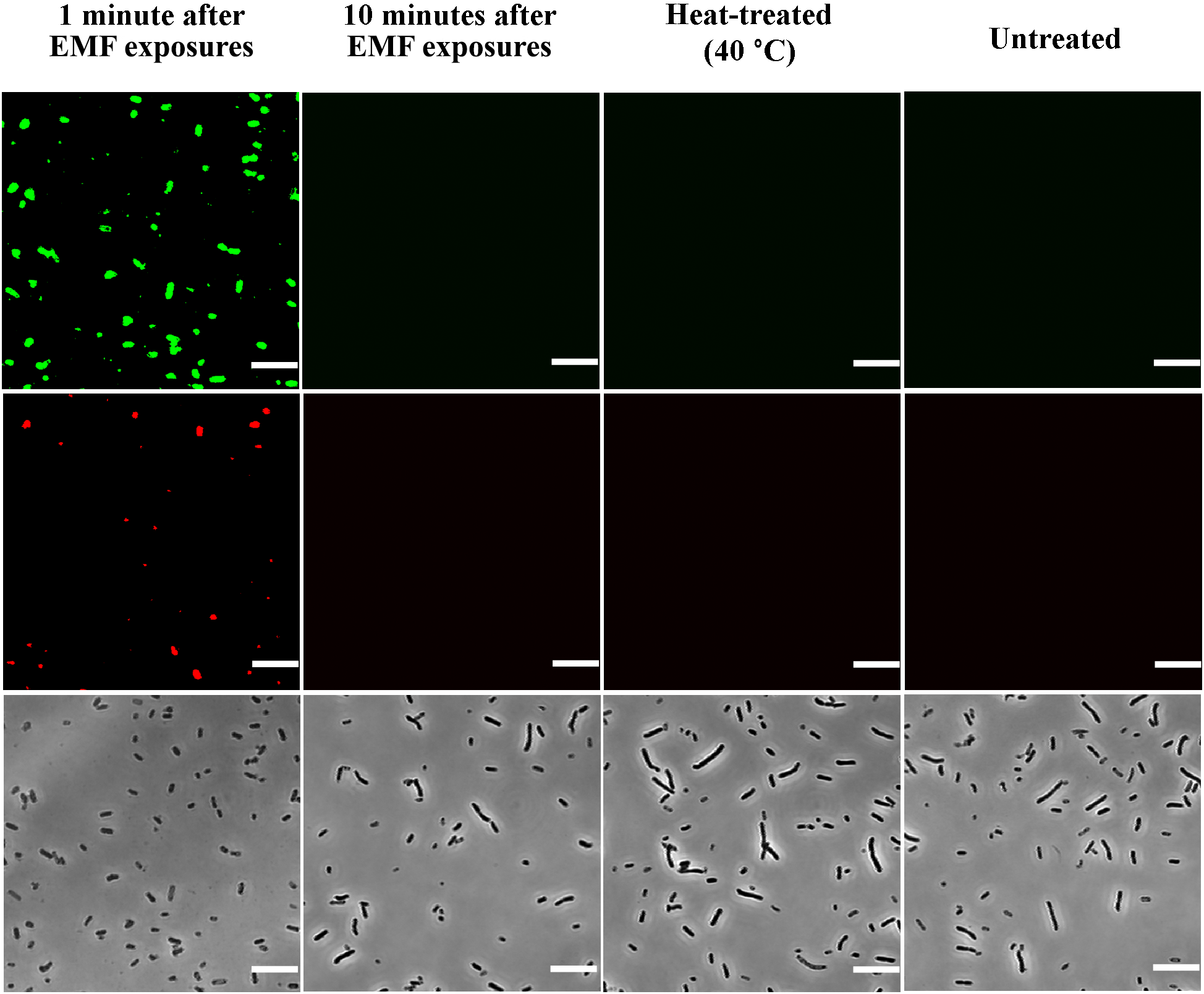


**SUPPLEMENTARY FIG. S3| Bacterial cells response to Peltier heat treatment and untreated cells.** SEM, CLSM and phase contrast images showing unchanged cell appearance with no nanosphere intake. Scale bars are 5 μm.


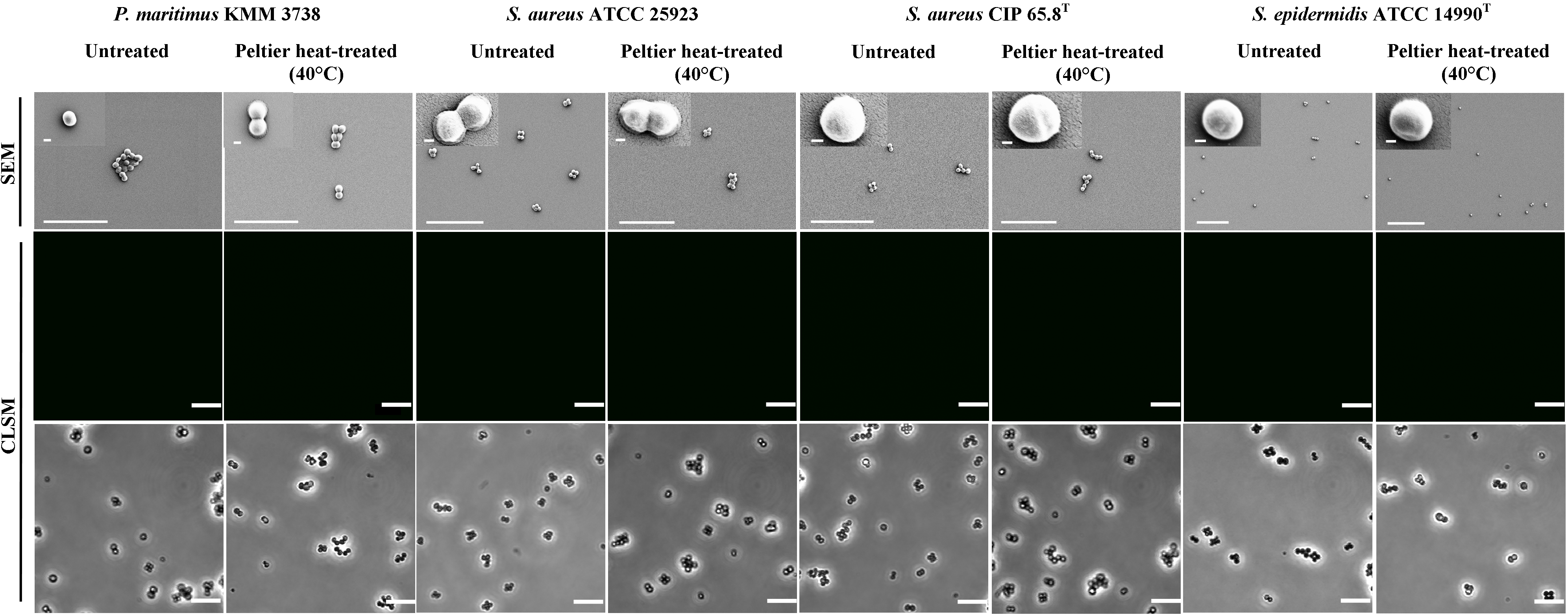


**SUPPLEMENTARY FIG. S4| No uptake of 23.5 nm nanospheres by the control groups.** Typical TEM images of thin-sectioned (80 nm) cells showing 23.5 nm nanospheres outside and around the cell membrane of untreated and heat treated cells and uniform cytosol with no nanospheres. Scale bars are 200 nm.

**
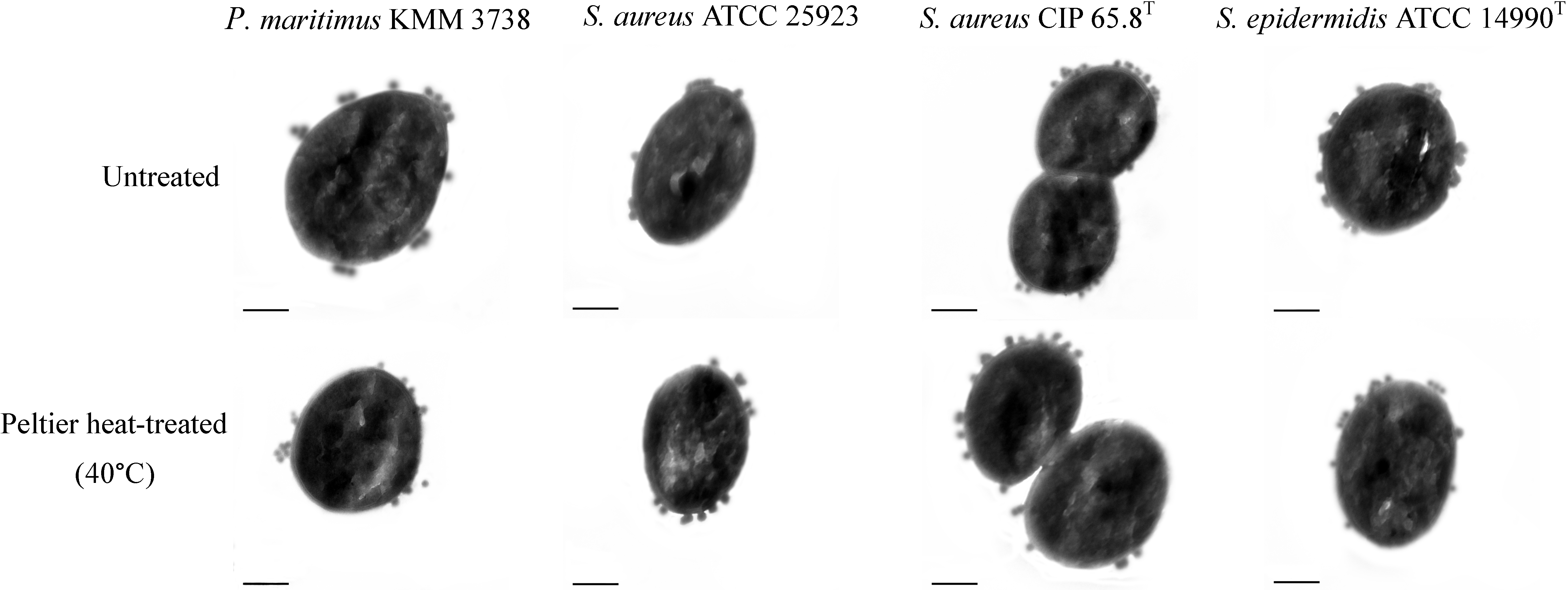
**

**SUPPLEMENTARY FIG. S5| The CST simulation parameters, which were used for the simulation.**


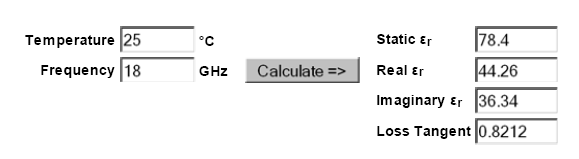


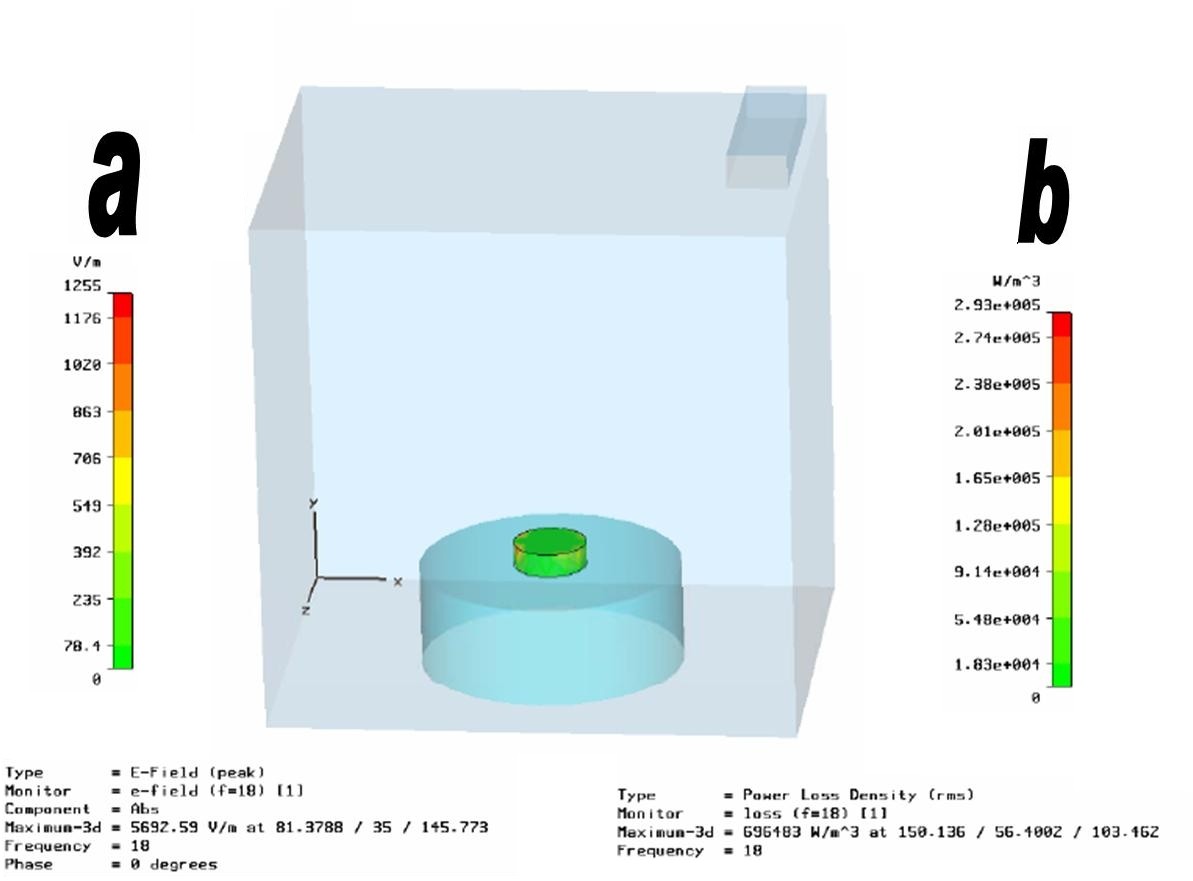

Supplement: Supplementary Information [file srep10980-s1.doc]
